# Ribosomal Protein 6 Phosphorylation Regulates Translational Responses to Dietary Restriction

**DOI:** 10.1093/geroni/igab046.2150

**Published:** 2021-12-17

**Authors:** Jarod Rollins

**Affiliations:** MDI Biological Laboratory, Salbury Cove, Maine, United States

## Abstract

Forms of dietary restriction like intermittent fasting (IF) and caloric restriction (CR) promote health and longevity through changes in gene expression. While the transcriptional changes that occur in response to DR have been well described across several species, the role of translational regulation has lagged. Using polysome profiling and mRNA-seq, we quantified changes in actively translated mRNAs that occur in C. elegans under CR compared to well-fed conditions. The analysis revealed hundreds of transcripts regulated on the translational level that would have been missed using conventual transcriptomics. Among the translationally down-regulated genes that where pro-longevity when knocked down were regulators of the cell-cycle: fbxb-24, sdz-33, kbp-1, and cdk-2. In search of the mechanisms regulating selective translation under CR we investigated a role for ribosomal protein 6 (RPS-6) as its phosphorylation status is thought to regulate cell cycle and selective translation of mRNA transcripts. Using RPS-6 phospho-null and phospho-mimetic mutants, we show that phosphorylation and de-phosphorylation of RPS-6 is necessary for the pro-longevity effects of CR and IF. Furthermore, we show that IF is more beneficial for retaining locomotion with age than CR and that endogenously tagged RPS-6 ::mCherry accumulates in body wall muscle under fasting. However, the benefit of IF on locomotion is lost in RPS-6 phospho-mimetic mutants. Together, results suggest that protein translation is enhanced in the muscle under IF to prevent sarcopenia in a way dependent on RPS-6. Translatome analysis of the phospho-mutant suggested a role for RPS-6 in selective translation of p38 mitogen-activated protein kinases.

